# Rechargeable Aqueous Zinc–Halogen Batteries: Fundamental Mechanisms, Research Issues, and Future Perspectives

**DOI:** 10.1002/advs.202305061

**Published:** 2023-11-08

**Authors:** Liaona She, Hao Cheng, Ziyan Yuan, Zeyu Shen, Qian Wu, Wei Zhong, Shichao Zhang, Bing Zhang, Chengwu Liu, Mingchang Zhang, Hongge Pan, Yingying Lu

**Affiliations:** ^1^ Institute of Science and Technology for New Energy Xi'an Technological University Xi'an 710021 P. R. China; ^2^ State Key Laboratory of Chemical Engineering Institute of Pharmaceutical Engineering College of Chemical and Biological Engineering Zhejiang University Hangzhou 310027 China; ^3^ ZJU‐Hangzhou Global Scientific and Technological Innovation Center Zhejiang University Hangzhou 311215 China; ^4^ Institute of Wenzhou Zhejiang University Wenzhou 325006 China; ^5^ Department of Chemical Engineering Shanghai Jiao Tong University Shanghai 200240 P. R. China

**Keywords:** aqueous batteries, bromine, chlorine, iodine, zinc–halogen batteries

## Abstract

Aqueous zinc–halogen batteries (AZHBs) have emerged as promising candidates for energy storage applications due to their high security features and low cost. However, several challenges including natural subliming, sluggish reaction kinetics, and shuttle effect of halogens, as well as dendrite growth of the zinc (Zn) anode, have hindered their large‐scale commercialization. In this review, first the fundamental mechanisms and scientific issues associated with AZHBs are summarized. Then the research issues and progresses related to the cathode, separator, anode, and electrolyte are discussed. Additionally, emerging research opportunities in this field is explored. Finally, ideas and prospects for the future development of AZHBs are presented. The objective of this review is to stimulate further exploration, foster the advancement of AZHBs, and contribute to the diversified development of electrochemical energy storage.

## Graphical abstract

In this review, first the fundamental mechanisms and scientific issues associated with aqueous zinc–halogen batteries (AZHBs) are summarized. Then the research issues and progresses related to the cathode, separator, anode, and electrolyte are discussed. Additionally, emerging research opportunities in this field are explored. Finally, ideas and prospects for the future development of AZHBs are presented.

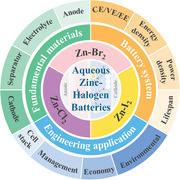

## 1 Introduction

The increasing demand for electrification has arisen as a response to the need for mitigating various environmental and energy‐related crises caused by fossil fuels.^[^ ^1^ ^]^ Consequently, electrochemical energy storage (EES) devices have gained significant attention as a potential solution. These devices find applications in numerous scenarios, such as grid‐scale energy storage, portable electronics, and electric vehicles.^[^ ^2^, ^3^ ^]^ Lithium‐ion batteries (LIBs) have dominated the EES market since their commercialization in 1991, owing to their excellent energy density and durable cycle life.^[^ ^4^ ^]^ However, it is important to note that LIBs based on intrinsic combustible organic electrolytes have raised safety concerns for energy storage applications.^[^ ^5^, ^6^ ^]^ There have been multiple incidents of LIBs explosions reported both domestically and internationally, involving various devices ranging from mobile phones to energy storage power stations.^[^ ^7^, ^8^ ^]^ These safety hazards highlight the need for continuous research and development to address and improve the safety aspects of EES.

**Figure 1:**
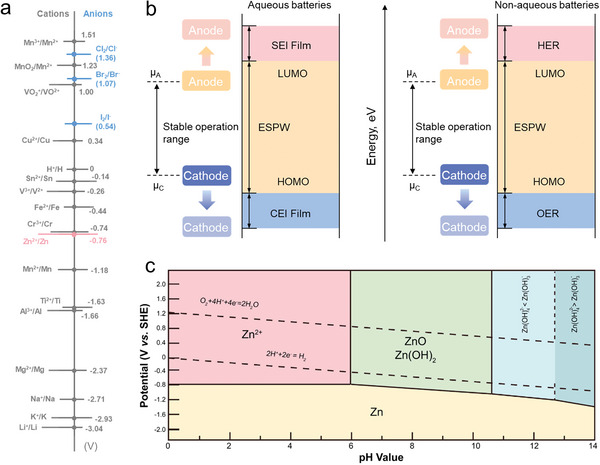
a) Standard redox potential of common electrode elements. b) Band diagrams of the HOMO and LUMO of batteries with nonaqueous electrolytes and aqueous electrolytes. c) Pourbaix diagram for Zn–H_2_O system.^[^ ^33^ ^]^ Reproduced with permission. Copyright 2021, Cell Press.

Aqueous metal batteries have garnered significant attention in recent years due to the inherent safety features of water‐based electrolytes.^[^ ^9^, ^10^ ^]^ While alkali metals possess extremely low redox potentials (**Figure** **1**a), their high reactivity with water poses challenges for battery stability, as they struggle to withstand the voltage window limitations of water (1.23 V).^[^ ^11^ ^]^ In contrast, multivalent metals with greater stability have gained popularity.^[^ ^12^ ^]^ Among them, zinc (Zn) metal has received considerable attention due to its suitable potential (−0.76 V vs standard hydrogen electrode (SHE)), higher mass/volume capacity (820 mAh g^−1^ and 5855 mAh cm^−3^), strong deposition/stripping kinetics, abundant reserves, and environmental friendliness.^[^ ^13^, ^14^, ^15^ ^]^ Investigation of Zn‐based batteries have been ongoing for over 200 years. One of the most representative event is the commercialization of alkaline Zn‐manganese dioxide disposable battery which has been widely used in small electronic devices.^[^ ^16^, ^17^, ^18^ ^]^ In recent years, researchers have discovered the excellent stripping/deposition characteristics of Zn metal under neutral/weakly acidic conditions, offering hope for its development as a rechargeable battery.^[^ ^19^, ^20^ ^]^ Transition metal oxides, such as manganese (Mn) and vanadium (V), have gained significant attention due to their high redox potential (Figure 1a) and high specific capacity.^[^ ^21^, ^22^, ^23^ ^]^ However, these layered cathodes face challenges such as cation dissolution, structural collapse, low conductivity, and complex reaction mechanisms.^[^ ^24^, ^25^, ^26^ ^]^ Exciting breakthroughs are still anticipated in overcoming these obstacles.^[^ ^27^, ^28^ ^]^ On the other hand, halogen (Cl_2_, Br_2_, and I_2_) based cathodes utilizing conversion reactions exhibit comparable redox potentials (Figure 1a) and significantly lower costs, enabling aqueous zinc–halide batteries (AZHBs) become promising candidates in EES devices.^[^ ^29^, ^30^ ^]^ Note that fluorine in its elemental form is a highly toxic and corrosive gas, and due to its natural state being gaseous, it poses significant challenges as a cathode.^[^ ^31^ ^]^ Therefore, it is not within the scope of discussion in this paper. Besides, due to its rarity and radioactive nature, astatine is also not within the scope of discussion in this paper.^[^ ^32^ ^]^

AZHBs indeed have a long research history and have made significant progress.^[^ ^33^, ^34^ ^]^ Among them, the development of Zn–Br_2_ batteries (ZBBs) has been particularly prominent, with successful demonstrations of megawatt‐scale applications.^[^ ^35^ ^]^ ZBBs were initially patented in 1885 and later developed as a hybrid flow system by Exxon, Gould, and National Aeronautics and Space Administration (NASA) in the 1970s. The battery exhibits a theoretical energy density of 440 Wh kg^−1^ and a theoretical output of 1.85 V.^[^ ^36^ ^]^ Despite significant progress in the field of ZBBs, challenges still persist with the Br_2_, including issues related to Br_2_ diffusion and the relatively low kinetics of the Br_2_.^[^ ^37^, ^38^ ^]^ Hence, current research focuses on exploring and developing electrolytes with strong complexation abilities, high‐performance ion‐conducting separators to minimize Br_2_ crossover, and high‐activity electrode materials to boost the kinetics of the Br_2_ in ZBBs.^[^ ^39^, ^40^ ^]^ Compared to the ZBBs, the practical feasibility of Zn–Cl_2_ batteries (ZCBs) is relatively lower due to the increased volatility and toxicity of chlorine gas, which raises concerns regarding safety, stability, and maintenance costs.^[^ ^41^, ^42^ ^]^ The first airship powered by a redox flow battery, weighing 435 kg, was powered by a ZCB system invented by Charles Renard in 1884.^[^ ^43^ ^]^ From a safety perspective, I_2_ is beneficial for the stability of batteries due to its higher boiling point. In 1949, Martin developed the Zn–I_2_ batteries (ZIBs) for academic demonstration, utilizing metallic Zn as anode and potassium iodide solution as the electrolyte.^[^ ^44^ ^]^ In 2015, Wang et al. demonstrated that by employing a near‐neutral electrolyte with 5.0 m ZnI_2_, flow ZIBs achieved a maximum discharge energy density of up to 167 Wh L^−1^, primarily due to the high solubility of the I^−^/ I_3_^−^ redox couple.^[^ ^45^ ^]^ These results confirmed the potential of ZIBs as a promising candidate, offering high energy density while avoiding the use of strong acids and corrosive components. Recently, AZHBs with flowless systems have been reported in addition to flow systems.^[^ ^46^ ^]^ These static systems offer advantages in battery structure, making them less cumbersome.^[^ ^47^ ^]^ In fact, whether it is a flow system or a static battery, the main scientific issues they face are actually highly similar.

In this review, we first summarize the fundamental mechanisms and scientific issues associated with AZHBs. We then discuss the research issues and progresses related to the cathode, separator, anode, and electrolyte. Additionally, we explore emerging research opportunities in this field. Finally, we present ideas and prospects for the future development of AZHBs. We hope this review will contribute to a deeper insight into the exploration and optimization of AZHBs and further promote their practical applications.

## 2 Fundamental Redox Chemistry of AZHBs

During the cycling process of Zn‐based batteries, spontaneous chemical reactions may occur when Zn metal and electrolyte come into direct contact,^[^ ^48^ ^]^ as shown in Figure 1b. While aqueous electrolytes do not produce a solid‐electrolyte interphase (SEI) film like nonaqueous electrolyte, they give rise to issues such as hydrogen evolution reactions (HERs).^[^ ^49^ ^]^ On the other hand, in organic electrolytes, the cathode material and electrolyte directly interact to form the cathode‐electrolyte interface (CEI), where oxygen evolution reactions (OERs) take place in aqueous electrolytes.^[^ ^50^, ^51^ ^]^ The electrochemical stable potential window (ESPW) plays a crucial role in determining the stability of electrolyte operation.^[^ ^52^ ^]^ Figure 1b illustrates that the ESPW corresponds to the energy difference between the lowest unoccupied molecular orbital (LUMO) and the highest occupied molecular orbital (HOMO), where µA and µ_C_ represent the electrochemical potentials of the anode and cathode, respectively.^[^ ^53^, ^54^, ^55^ ^]^ In theory, increasing the potential difference between the cathode and anode can enhance the output voltage.^[^ ^56^ ^]^ However, if µA exceeds the LUMO energy, it will result in HERs and if µ_C_ is lower than the HOMO energy, it will lead to OERs.^[^ ^57^ ^]^ Therefore, the regulation of electrolyte mainly aims to expand the ESPW, thereby promoting the operating voltage.

Figure 1c displays the Pourbaix diagram of Zn metal, which illustrates the energy storage mechanism of Zn through the reversible electrodeposition/dissolution process.^[^ ^58^ ^]^ Being an amphoteric metal, the mechanism and equilibrium potential of Zn electrode reactions are strongly influenced by the electrolyte environment.^[^ ^59^ ^]^ The hydrolysis of Zn^2+^ in electrolytes with varying pH leads to the formation of Zn(OH)_2_, Zn(OH)_3_^−^, and Zn(OH)_4_^2–^. The Pourbaix diagram can be constructed based on the Nernst equation

(1) $${\mathrm{Zn}}^{2+}+2e^{-}↔{\mathrm{Zn}}^{0},E_{1}=-0.762+0.0295lg\left[{\mathrm{Zn}}^{2+}\right]$$

(2) $$\mathrm{Zn}{\left(\mathrm{OH}\right)}_{2}+2e^{-}↔{\mathrm{Zn}}^{0}+2OH^{-},E_{2}=-0.439-0.0591\;\mathrm{pH}$$

(3) $$\begin{array}{ccc} & & \mathrm{Zn}{\left(\mathrm{OH}\right)}_{3}^{-}+2e^{-}↔{\mathrm{Zn}}^{0}+3OH^{-},\\ & & E_{3}=-0.0886\;\mathrm{pH}+0.0295\mathrm{lg}[\mathrm{Zn}{\left(\mathrm{OH}\right)}_{3}^{-}] \end{array}$$

(4) $$\begin{array}{ccc} & & \mathrm{Zn}{\left(\mathrm{OH}\right)}_{4}^{2-}+2e^{-}↔{\mathrm{Zn}}^{0}+{4\mathrm{OH}}^{-},\\ & & E_{4}=-0.1182\;\mathrm{pH}+0.0295\;\mathrm{lg}\left[\mathrm{Zn}{\left(\mathrm{OH}\right)}_{4}^{2-}\right] \end{array}$$

These reactions allow us to divide the Pourbaix diagram into four regions, representing different thermodynamically stable reactants involved in the reversible deposition/dissolution process.^[^ ^60^ ^]^ Since the [Zn] in various aqueous Zn‐based batteries (AZBs) may be different, the thermodynamically stable pH range of each reactant could be roughly given as follows: 1) pH < 8, primarily Zn^2+^ in the aqueous electrolyte, 2) 8 ≤ pH ≤ 12, the reactants are mainly Zn(OH)_2_ and Zn(OH)_3_^−^, 3) pH > 12, Zn(OH)_4_^2−^ in the alkaline electrolyte. Indeed, the presence of various anions can have a slight influence on the pH values of the reactants in the Pourbaix diagram.^[^ ^33^ ^]^ These anions can cause slight modifications to the pH ranges at which the reactants are thermodynamically stable. The specific effect depends on the interaction between the anions, the Zn species, and the hydroxide ions. As a result, the pH ranges mentioned earlier may exhibit slight variations when different anions are present in the electrolyte.

Halogen elements as the cathode represent a typical conversion‐type, which have been developed in the past decade.^[^ ^61^, ^62^ ^]^ A typical conversion reaction of halogen elements in AZHBs can be described as following

(5) $${\mathrm{nX}}^{0}+ne^{-}↔{\mathrm{nX}}^{-}$$

where X is typically Cl, Br, and I, the battery operation is driven by the reversible conversion reaction between X^0^ and X^−^, accompanied by electron transfer.^[^ ^47^ ^]^ As a result, a superior long‐term cyclic life can be obtained due to less lattice distortion, especially for multivalent‐ions systems.^[^ ^63^ ^]^

Cl^−^ possess several attractive advantages, including i) high theoretical volumetric energy density (2500 Wh L^−1^) and gravimetric energy density (1100 Wh kg^−1^),^[^ ^64^ ^]^ ii) wide electrochemical stability window owing to its high electronegativity,^[^ ^65^ ^]^ and iii) low‐cost and dendrite‐free formation during cycling.^[^ ^66^ ^]^ Taking advantage of these benefits, the ZCBs have been developed, demonstrating a relatively high cell voltage of 2.12 V.^[^ ^67^ ^]^ The overall electrode reactions are described as follows

(6) $$\mathrm{Anode}:Zn^{0}↔{\mathrm{Zn}}^{2+}+2e^{-}$$

(7) $$\mathrm{Cathode}:Cl_{2}+2e^{-}↔2Cl^{-},E^{0}=+1.36\;V\;\mathrm{versus}\mathrm{SHE}$$

(8) $$\mathrm{Overall}:Cl_{2}+\mathrm{Zn}↔{\mathrm{ZnCl}}_{2},E_{\mathrm{cell}}=2.12\;V$$

To date, only a few studies have reported ZCBs system. Chen et al. demonstrated an aqueous ZCB modulated by MnO_2_, where MnO_2_ act as redox adsorbent to trap Cl_2_ in the cathode, thereby reducing the generation of Cl_2_ in battery.^[^ ^66^ ^]^ Additionally, first‐principles calculations revealed that carbon felt deposited with MnO_2_ exhibits a stronger adsorption tendency for Cl_2_ (−4.93 eV) compared to bare carbon felt (−0.05 eV), minimizing the occurrence of chlorinated by‐products. As a result, the Zn–Cl_2_@MnO_2_ battery achieved a high discharge voltage of 2.0 V at 2.5 mA cm^−2^, and maintained an average Coulombic efficiency (CE) of 91.6% after 1000 cycles. However, rechargeable ZCBs are still in the early stage of basic research and development, further development of ZCBs is still required on practical applications.

Compared to ZCBs, ZIBs systems are becoming increasingly popular among researchers due to the abundant reserves, low cost, and good compatibility with electrolytes.^[^ ^31^ ^]^ In the typical cycling process of ZIBs, the I_2_ content of the cathode plays a significant role in the electrochemical reaction.^[^ ^68^ ^]^ On the cathode side, an adequate supply of iodine is present, resulting in the loss of electrons by I^−^ to generate slightly soluble I_2_.^[^ ^69^ ^]^ The I_2_ then spontaneously forms highly soluble I_3_^−^ by bonding with excess I^−^ ions (stage I and II). As the iodine content decreases, only I^−^ ions are oxidized to insoluble iodine (stage III).^[^ ^68^ ^]^ During the electrochemical process, the universal generation of various polyiodides (I_3_^−^, I_5_^−^, I_7_^−^, and I_9_^−^) occurs, leading to the formation of different intermediates based on the thermodynamics experienced.^[70]^ Reverse reactions occur during discharging. The electrode reactions and overall reactions are described as follows

(9) $$\mathrm{Anode}:Zn^{0}↔{\mathrm{Zn}}^{2+}+2e^{-}$$

Cathode:

(10) $$\begin{array}{ccc} & & \mathrm{Stage}\;I:\;I_{3}^{-}+2e^{-}↔I^{-}+I_{2}+2e^{-}↔3I^{-}+2e^{-},\\ & & \;\;\;\;E^{0}=+0.536\;V\;\;\mathrm{versus}\;\;\mathrm{SHE} \end{array}$$

(11) $$\mathrm{Stage}\;\mathrm{II}:\;3I_{2}+2e^{-}↔2I_{3}^{-},E^{0}=+0.789\;V\;\mathrm{versus}\;\;\mathrm{SHE}$$

(12) $$\mathrm{Stage}\;\mathrm{III}:\;I_{2}+2e^{-}↔2I^{-},E^{0}=+0.621\;V\;\mathrm{versus}\;\;\mathrm{SHE}$$

Overall:

(13) $$I_{3}^{-}+\mathrm{Zn}↔{\mathrm{Zn}}^{2+}+3I^{-},E_{\mathrm{cell}}=1.299\;\;\;V$$

(14) $$3I_{2}+\mathrm{Zn}↔{\mathrm{Zn}}^{2+}+2I^{-},E_{\mathrm{cell}}=1.552\;\;V$$

(15) $$I_{2}+\mathrm{Zn}↔{\mathrm{Zn}}^{2+}+2I^{-},E_{\mathrm{cell}}=1.384\;\;\;V$$

Indeed, polyiodide species in ZIBs often exhibit poor reversibility, which can significantly impact the actual energy density of the batteries. To exacerbate matters, polyiodide ions are highly prone to shuttle to the anode, causing shuttle effects that can damage the battery's CE and result in self‐discharge.^[^ ^71^, ^72^ ^]^ Additionally, at higher charging states and/or lower temperatures, polyiodide ions have a tendency to dissociate, releasing insoluble I_2_.^[^ ^73^, ^74^ ^]^ This phenomenon can lead to blockages in the battery system's channels. These issues pose significant challenges to the practical implementation of ZIBs, as they can substantially reduce the overall performance and cycling stability of the batteries.^[^ ^75^ ^]^

Among the halogen elements, ZBBs have seen the most successful commercialization in recent years. The incorporation of Br_2_ into AZBs, whether in redox flow batteries or static batteries, offers several advantages, including high energy density, high cell voltage, and low cost.^[^ ^65^, ^66^, ^76^ ^]^ The Br_2_/Br^−^ redox couple is utilized as the active substance in the cathode, and the reaction can be described as follows

(16) $$\mathrm{Anode}:\;{\mathrm{Zn}}^{0}↔{\mathrm{Zn}}^{2+}+2e^{-}$$

Cathode:

(17) $${\mathrm{Br}}_{2}+2e^{-}↔2Br^{-},E^{0}=+1.065\;V\;\mathrm{versus}\;\;\mathrm{SHE}$$

(18) $${\mathrm{Br}}_{2}+Br_{m}^{-}↔{\mathrm{Br}}_{m+2}^{-}$$

(19) $${\mathrm{Br}}_{m+2}^{-}+\left(m+1\right)e^{-}↔\left(m+2\right){\mathrm{Br}}^{-}$$

(20) $$\mathrm{Overall}:\;{\mathrm{Br}}_{2}+\mathrm{Zn}↔{\mathrm{ZnBr}}_{2},E_{\mathrm{cell}}=1.828\;\;V$$

Reactions (18) and (19) illustrate that bromine molecules formed through the oxidation of bromide anions, can undergo further reactions to generate polybromide anions such as Br_3_^−^, Br_5_^−^, and Br_7_^−^ at the cathode.^[^ ^68^, ^77^ ^]^ In aqueous electrolytes, the presence of polybromides and elemental bromine can lead to their diffusion toward the Zn anode, causing electrode corrosion, as well as the shuttle effect like in ZIBs.^[^ ^78^ ^]^

**Figure 2:**
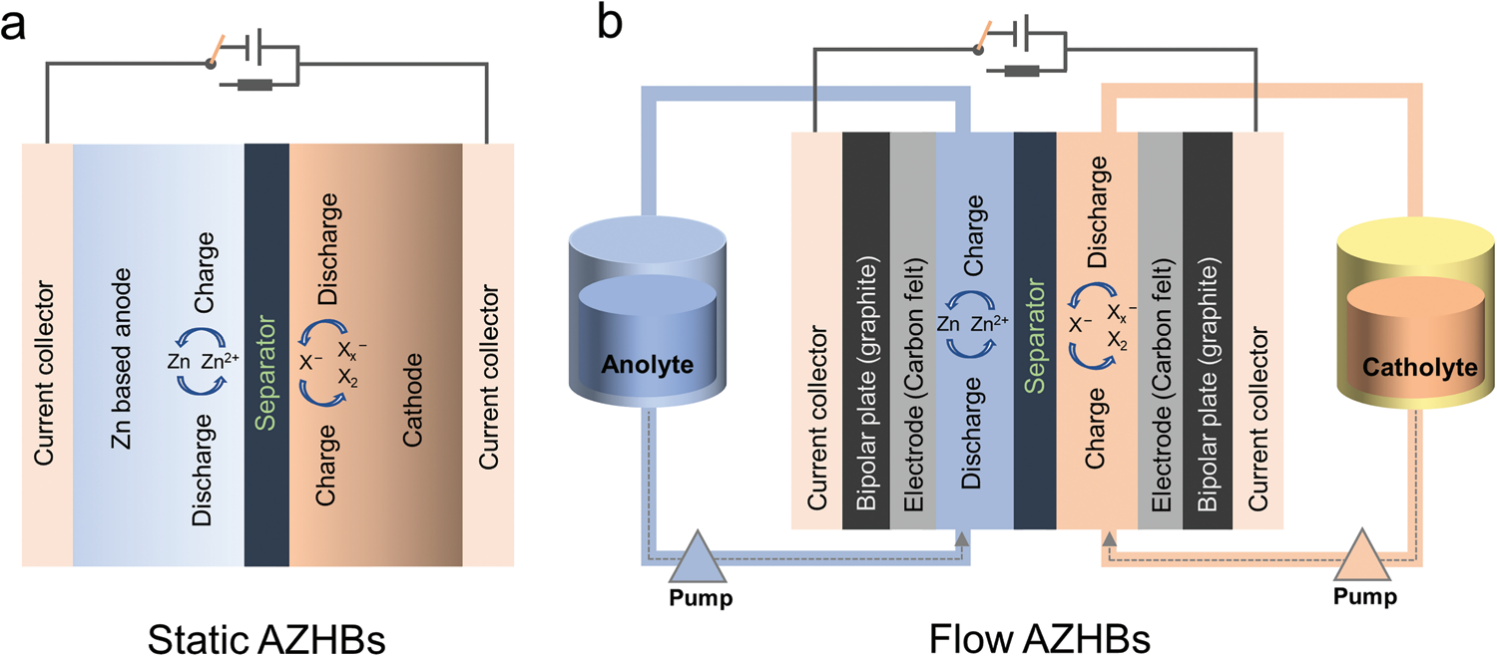
The schematic representations of a) a static AZHBs, b) a traditional flow AZHBs. X represents halogen.

AZHBs can be classified into two types based on their structure (**Figure** **2**): static systems and flow systems (dual flow and single flow).^[^ ^79^ ^]^ Each system has its advantages and suitable applications. The liquid flow configuration offers the following advantages: i) Uniform mixing: active species can be effectively mixed through stirring or circulating flow, which helps maintain reaction uniformity, promote reaction rates, and improve overall efficiency.^[^ ^47^ ^]^ ii) Temperature control: fluid systems allow for easier control of reaction temperature. By adjusting the flow rate and temperature of the cooling or heating medium, the desired temperature conditions can be achieved.^[^ ^80^ ^]^ iii) Continuous operation: fluid systems enable continuous operation, with a steady supply of reactants and continuous collection of products, resulting in highly efficient reaction processes.^[^ ^81^ ^]^ iv) Energy and power can be independently designed, mainly targeting nondeposition reactions on the cathode side.^[^ ^75^, ^82^ ^]^ On the other hand, static configuration offers the following advantages: i) Ease of operation: static systems are simpler compared to fluid systems, as they do not require stirring equipment or circulation systems, making them easier to operate.^[^ ^83^ ^]^ ii) Lower equipment costs: static systems have relatively lower equipment costs since they do not require complex flow devices.^[^ ^84^, ^85^ ^]^ They are suitable for smaller‐scale laboratory or industrial applications. **Table** **1** compares the representative achieved parameters of different AZHBs. Of note, both flow batteries and static batteries face similar scientific challenges. In the following sections, we will summarize and discuss the scientific challenges and research progress of AZHBs.

**Table 1:** The key parameters of comparison of AZHBs.

Type		Redox couple	Cathodic reaction state	Working potential [V]	Energy density [Wh L^−1^]	Power density [W L^−1^]	Energy efficiency [%]	Life	Refs.
ZBBs	Flow	Br_2_/Br^−^	Liquid	1.85	60–90	40	60–75	2000 cycles	[86]
	Static		Liquid	1.8	12.2	–	80	1000 cycles	[87]
ZIBs	Flow	I_3_^−^/I^−^	Liquid	1.28	322	99	90	40 cycles	[45]
	Static		Liquid	1.30	320	80	89	10 000 cycles	[88]
ZCBs	Flow	Cl_2_/Cl^−^	Gas	2.12	–	–	66	20 cycles	[89]
	Static		Gas	2.60	194	–	–	1000 cycles	[90]

## 3 Current Researches on Critical Components of AZHBs

**Figure 3:**
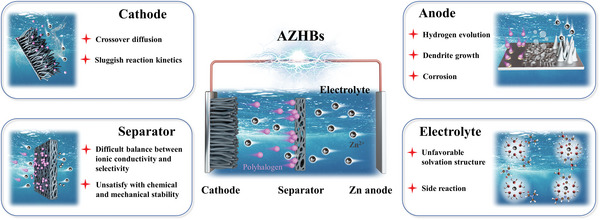
Schematic illustration of challenges faced by key components of AZHBs.

AZHBs have garnered significant attention as promising energy storage solutions. However, they encounter several substantial challenges that must be effectively addressed to facilitate their widespread adoption. These challenges can be categorized into four fundamental areas (**Figure** **3**):

Cathode. i) Crossover diffusion: A primary challenge in AZHBs is the crossover diffusion of polyhalogen species from the cathode to the anode compartment. This phenomenon can result in the loss of active materials and a reduction in CE. ii) Sluggish reaction kinetics: AZHBs face issues related to sluggish reaction kinetics in cathodic processes, particularly concerning the reduction of halogen species. These kinetics‐related challenges lead to lower power output and slower charge/discharge rates, thereby limiting their applicability in high‐power applications.

Anode. i) Hydrogen evolution: AZHBs are susceptible to hydrogen evolution reactions at the anode due to limitations of ESPW, which can detrimentally affect overall CE and safety. ii) Dendrite growth: The formation and growth of Zn dendrites on the anode surface pose a significant challenge. Dendrite formation can lead to short circuits and reduced cycle life. iii) Corrosion: Anode corrosion can occur due to side reactions, resulting in a reduced lifespan for the anode and overall system degradation.

Electrolyte. i) Unfavorable solvation structure: The solvation structure of Zn^2+^ in the electrolyte can be unfavorable, leading to issues such as poor ion mobility and low conductivity, which in turn hinder battery performance. ii) Side reactions: Undesirable side reactions can occur in the electrolyte due to the thermodynamic instability of water and Zn in the electrolyte.

Separator. i) Difficult balance between ionic conductivity and selectivity: Selecting an appropriate separator material presents a challenge, as it necessitates striking a delicate balance between high ionic conductivity to facilitate ion transport and adequate selectivity to prevent the crossover of halogen species. ii) Unsatisfactory chemical and mechanical stability: The separator must maintain its chemical and mechanical stability throughout the battery's operational lifetime, even in the presence of corrosive halogens.

Effectively addressing these challenges through innovative materials, design enhancements, and advanced engineering solutions is imperative for the successful development and deployment of AZHBs across various applications, ranging from stationary energy storage to grid integration.

To achieve efficient and reliable AZHBs, it is crucial to achieve the following mission principles: 1) Fulfillment of high energy efficiency (EE) and power density.^[^ ^71^ ^]^ 2) Achievement of long cycle life and excellent capacity retention.^[^ ^91^ ^]^ 3) Utilization of cost‐effective materials and manufacturing processes.^[^ ^92^ ^]^ The electrodes, electrolytes, and separators are the core components of the battery and play a crucial role in determining the electrochemical performance of AZHBs.^[^ ^33^, ^93^ ^]^ In recent years, researchers have conducted extensive studies focusing on these areas to enhance the performance of AZHBs. In the following section, we will analyze these details in‐depth.

### 3.1 Cathodes

For the cathode, halogens such as Br_2_ and I_2_ are commonly subjected to sluggish reaction kinetics and polyanion shuttle diffusion issues. Currently, the main approach to address these problems is to modify them through the construction of cathode host materials. Depending on the different material properties, the modified cathode materials can be classified into the following categories:

#### 3.1.1 Carbon‐Based Materials

**Figure 4:**
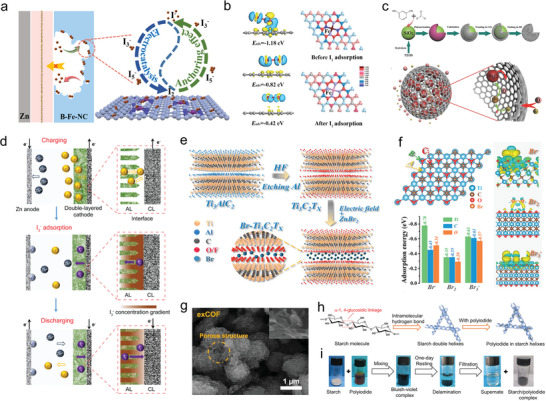
a) Schematic illustration for the proposed reaction mechanism of polyiodide adsorption/conversion on the matrix of B−Fe−NC. b) Charge density difference of the optimized configurations of Fe−N_4_−carbon (Fe−N_4_−C). Reproduced with permission.^[^ ^103^ ^]^ Copyright 2022, American Chemical Society. c) Schematic illustration to the fabrication of the cage‐like porous carbon and its capture Br_2_ complex. Reproduced with permission.^[^ ^104^ ^]^ Copyright 2016, Wiley‐VCH. d) Schematic illustrations of the operation mechanisms of a ZIB with a double‐layered cathode (AL in green and CL in black). Reproduced with permission.^[^ ^110^ ^]^ Copyright 2021, American Chemical Society. e) Schematic illustration of the Br−Ti_3_C_2_T*_x_* design and fabrication. f) DFT simulation about the adsorption energy of Br species on Ti_3_C_2_T*_x_* MXene flakes and optimized charge‐of‐density patterns of Br species around Ti adsorption sites. Reproduced with permission.^[^ ^111^ ^]^ Copyright 2021, American Chemical Society. g) SEM images of Br_2_‐exCOF electrode. Reproduced with permission.^[^ ^112^ ^]^ Copyright 2023, Elsevier. h) The molecule structure of starch polymer chains and the double‐helix structure of starch. i) The processes of the polyiodide capture by starch. Reproduced with permission.^[^ ^113^ ^]^ Copyright 2022, American Chemical Society.

Carbon‐based materials, such as carbon felts, carbon papers, carbon fiber, possess good conductivity, and large surface area owing to the intrinsically porous structure.^[^ ^75^, ^94^, ^95^, ^96^, ^97^, ^98^ ^]^ However, the finite interaction between the nonpolar carbons and polar polyiodides or polybromide is insufficient to inhibit the dissolution and shuttling effect of the active materials.^[^ ^99^ ^]^ To address this issue, heteroatom doping is an effective strategy for improving the stability and active material loading in the carbon‐based hosts by increasing the polar surface, thus providing more active sites for the host–guest affinity toward polyhalogen anion.^[^ ^25^, ^27^, ^88^, ^100^, ^101^, ^102^ ^]^ **Figure** **4**a illustrates the atomic bridging structure of metal‐nitrogen‐carbon (B‐Fe‐NC) proposed by Zhang et al., which not only improves the anchoring capacity, but also enhances the electrocatalytic redox conversion of iodine.^[^ ^103^ ^]^ Density functional theory (DFT) calculations, shown in Figure 4b, elucidate the differential adsorption of I_2_ molecules on Fe‐N_4_‐C, N‐C, and pure carbon surfaces. The unique atomic bridging structure of Fe‐N_4_‐C displays the strongest I_2_ adsorption energy of −1.18 eV, which is higher than that of N‐C (−0.82 eV), and pure C (−0.42 eV). Li et al. design a cage‐like porous carbon (CPC, Figure 4c) with specific pore structure via template method and entrapping capability of Br_2_ complex, which improve the electrode activity, voltage energy (VE), and power density.^[^ ^104^ ^]^ The microstructures of carbon‐based materials also play a role in their ability to immobilize iodine species. Generally, host materials with a larger surface area, higher micro/mesopore ratio, and closer stacking capability provide better adsorption.

The binding strength of the electrode substrate to the active material competes with the tendency of the active material to dissolve in the electrolyte.^[^ ^87^, ^98^, ^105^ ^]^ For instance, polyiodides exhibit high solubility in aqueous media, posing higher binding requirements on the substrate.^[^ ^106^, ^107^ ^]^ Traditional porous materials are often employed as conductive carriers, and iodine is loaded onto them through simple physical adsorption, chemical vapor deposition (CVD), or impregnation methods.^[^ ^30^, ^108^ ^]^ The interactions between the carrier and iodine primarily occur at the physical level, mainly through the adsorption of active iodine species on micropores or mesopores.^[^ ^109^ ^]^ It can be foreseen that exploring ways to enhance the affinity of the conductive substrate for halogens and even simultaneously catalyzing and improving the reaction kinetics will be the focus of future research.

#### 3.1.2 Organic Polymers

Compared to nonpolar carbon materials, the introduction of organic polymers such as polypyrrole (PPy), polyaniline, and poly (3, 4‐ethylenedioxythiophene) shows promise as candidates for AZHBs because they allow both physical and chemical interaction with polyiodide or polybromide. Li et al. construct a double‐layered cathode configuration, using carbon cloth as the conductive layer and polypyrene as the adsorptive layer (CC‐PPy).^[^ ^110^ ^]^ As shown in Figure 4d, when I^−^ in the electrolyte transfer electrons to the CC layer, they spontaneously form adsorbed I_3_^−^ at the CC‐PPy interface. These I_3_^−^ ions subsequently diffuse into the PPy layer under a concentration gradient, re‐exposing the interfacial adsorption sites for the newly formed I_3_^−^. During discharge, the dominant I_3_^−^ are reduced at the CC‐PPy interface to regenerate I^−^, effectively suppressing the shuttling of the I_3_^−^. This new cathode for static ZIBs achieved a high CE of 95.6% and VE of 91.3%. He et al. proposed an anion conducting polymer containing a poly (biphenyl pyridine) backbone with side chains of hydrophilic (2‐bromoethyl)trimrthylammonium bromide and hydrophobic hexyl bromide (PBP‐BTAB‐HB) to function as the bromide conductor and polybromide confiner.^[^ ^114^ ^]^ During charge process, soluble polybromide species are effectively confined within the electrode, inhibiting polybromide shuttling and ensuring excellent reversibility and stability for the bromide/polybromide redox reaction. This achieves high CE, VE, and EE of 92%, 81%, and 74%, respectively. To date, several materials such as carbonized tubular polypyrene (CTPPy), *N*, *N*’‐dimethyl‐1,3‐propanediamine grafted, triethylenetetramine‐crosslinked acrylic fiber/iodine (GC‐PAN/I), polyvinylpyrrolidone (PVP), and polymer polyaniline (PANI) have been proved effective in absorption of polyhalogen anion.^[^ ^106^, ^115^, ^116^, ^117^ ^]^ The combination of PVP and I_2_ can create soluble PVP‐I_3_^−^ complexes that suppress shuttle effect via reducing vapor pressure and restraining the dissolution of iodine species, respectively.^[^ ^116^ ^]^ PANI with positively charged nitrogen sites takes advantage of fast charge transfer and effectively immobilize polyiodide anions through chemical interaction, thus exhibiting high CE of 99.2% and excellent capacity retention.^[^ ^106^ ^]^

#### 3.1.3 Other Materials

In addition to polymers, metal electrocatalysts can also play a role in boosting the reaction kinetics and inhibiting the shuttle effect by providing electrocatalytic sites and immobilizing active species.^[^ ^72^, ^85^, ^105^ ^]^ Zhi et al. reported the electrodeposition of free Br^−^ ions into a Ti_3_C_2_T*_x_* MXene host (Br‐Ti_3_C_2_T*_x_*), as shown in Figure 4e.^[^ ^111^ ^]^ Due to the natural affinity between MXenes and Br species, Br^−^ ions can spontaneously anchor at the interface of the Br‐Ti_3_C_2_T*_x_* cathode, accompanied by rapid electron migration. DFT simulations (Figure 4f) demonstrated the direct redox of the Br^0^/Br^−^ couple and the confinement effect of the Br‐Ti_3_C_2_T*_x_* cathode, effectively mitigating the shuttle effect. As a result, the Br‐Ti_3_C_2_T*_x_* electrode exhibited a 81% capacity retention after 2000 cycles.

Constructing conductive hosts derived from covalent organic frameworks (COFs) and metal–organic frameworks (MOFs) is another approach to address solubility issues and sluggish electrochemical redox kinetics, utilizing the chemical‐interaction strategy.^[^ ^118^ ^]^ A 2D keto‐enamine linked exfoliated covalent organic framework (exCOF) with a porous structure (Figure 4g) exposes more active units, and its abundant functional groups can immobilize soluble bromide through physical confinement and chemical adsorption.^[^ ^112^ ^]^ In situ Raman spectra‐electrochemistry demonstrated the chemical structure variation of Br_2_‐exCOF, with an increased C═O peak at 1140 cm^−1^ during the charge process, indicating the intercalation of polybromide into the COF structure. It is speculated that the C─O bond accelerates the directional conversion of Br species. During the discharging process, the vibration region of C─O gradually decays, indicating the reduction of bromine elements on the COF to Br‐ions.

Qiao et al. proposed the use of starch as a host material for iodine adsorption to suppress the shuttle effect in ZIBs (Figure 4h), thereby enhancing their cycling lifespan.^[^ ^113^ ^]^ Through a series of evidence, it was demonstrated that multiple iodine ions can be embedded into the double helix structure of starch, leveraging the unique molecular structure of starch to enhance the adsorption capacity of multiple iodine ions. This highlights the role of starch in “structurally confined” iodine. Based on this strategy, ZIBs achieved CE close to 100% at a current density of 0.2 A g^−1^, as well as outstanding cycling lifespans of 10 000 cycles at 2 A g^−1^ and 50 000 cycles at 10 A g^−1^.

Generally, the introduction of polar materials with physical adsorption and chemical interaction can immobilize bromine/iodine species, enabling the long‐term stability of AZHBs. Carbon‐based materials with physical adsorption provide a conductive network that promotes faster kinetics, but their insufficient interaction may not completely prevent the shuttle of polyiodides.^[^ ^119^ ^]^ Polymers with chemical interaction offer stronger affinity between bromine/iodine species and the host. However, the low conductivity of polymers limits their further application.^[^ ^120^ ^]^ An ideal solution is to design conductive adsorption catalytic hosts that possess three key efficiency features, working synergistically to improve the reaction kinetics of halogen cathodes.

### 3.2 Separators

Separators play a vital role in AZHBs and significantly impact their overall performance. Traditionally, membranes serve to prevent the crossover of redox‐active species and facilitate the transfer of charge carriers within the circuit.^[^ ^121^, ^122^ ^]^ An ideal membrane for AZHBs should fulfill the following requirements:

- High selectivity: To prevent crossover and reduce capacity decay, the membrane should exhibit high selectivity toward active species, contributing to high CE.
- Ion conductivity: A battery with high ion conductivity will have low internal resistance, leading to high VE.
- Long‐term cycling performance: Chemical and mechanical stability are critical for ensuring the long‐term cycling performance of the battery.

Currently, two main types of membranes are used in AZHBs: ion‐exchange membranes (IEMs) and porous membranes. IEMs contain ion‐exchange groups responsible for ion transfer, while porous membranes achieve separation of active species and charge‐carrier ions through pore‐size exclusion.

**Figure 5:**
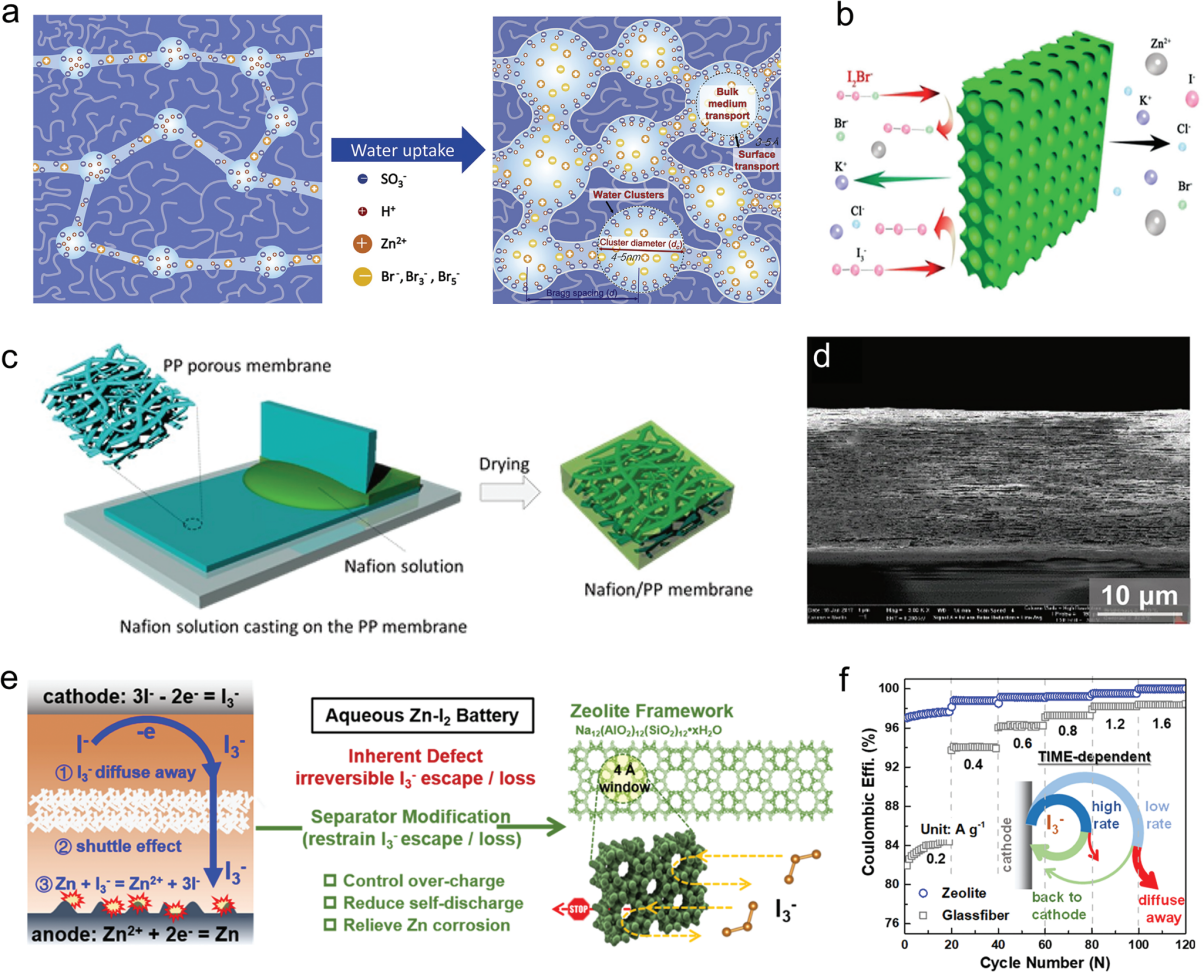
a) Proposed water cluster structures at a different hydration level. Reproduced with permission.^[^ ^126^ ^]^ Copyright 2018, Elsevier. b) Porous polyolefin membrane of ZIBs. Reproduced with permission.^[^ ^135^ ^]^ Copyright 2018, Wiley‐VCH. c) Schematic of the Nafion/PP membrane fabrication process. d) SEM images of cross‐section of the Nafion/PP membrane. Reproduced with permission.^[^ ^133^ ^]^ Copyright 2017, Nature Publishing Group. e) Fabrication of zeolite membrane and proposed working mechanism in ZIBs. f) CE collected at various current densities in ZIBs assembled with glass fiber separator (gray traces) and zeolite modified separator (blue traces). The rate‐dependent CE variation tendency is illustrated in the inset. Reproduced with permission.^[^ ^134^ ^]^ Copyright 2022, American Chemical Society.

Nafion membranes, as a type of IEM with a dense structure, exhibit high blocking ability against polyhalogen anion compared to porous membranes.^[^ ^123^, ^124^ ^]^ Lai et al. conducted a comparison between Nafion 115 and microporous membranes for a flow ZBB.^[^ ^125^ ^]^ While Nafion 115 membrane showed higher CE than the porous membrane, the membrane resistance of Nafion led to a 12% reduction in VE. Hydrated Nafion membranes have a nanophase separated morphology, featuring small water clusters surrounded by sulfonic acid groups of the Nafion polymer. These interconnected water clusters act as ion conducting channels, resulting in exceptionally ionic conductivity. The hydration state of Nafion membranes influences their electrochemical, transport, and mechanical properties. Increasing the water content leads to larger water clusters and higher ionic conductivity. In normal hydrated Nafion membranes, the narrow water cluster channel due to low hydration limits the permeation of negative ions through the water cluster. However, Kim et al., as displayed in **Figure** **5**a, pretreated Nafion membranes with prehydration at various temperatures, expanding the water cluster channels and creating a bulk liquid electrolyte medium‐like environment.^[^ ^126^ ^]^ This treatment weakened the influence of fixed negative charges and facilitated ionic conductivity.

Porous membranes, compared to IEMs, have gained increased attention due to their high ionic conductivity, high stability, and low cost.^[^ ^127^, ^128^ ^]^ A typical porous membrane can be prepared using the phase‐inversion method, comprising a bottom porous substrate and a top dense skin layer. The skin layer provides ion selectivity, while the porous support ensures mechanical and chemical stability of the membrane. Xie et al. fabricated an inexpensive polyolefin porous membrane that effectively blocked the migration of polyhalogen anions, reducing the shuttle effect (Figure 5b).^[^ ^129^ ^]^

One common issue with membranes is the trade‐off between ion selectivity and conductivity, where the former is related to CE and the latter is related to VE. In the case of IEMs, increasing ion conductivity requires higher ion‐exchange capacity. However, this leads to enhanced swelling of the membranes, enlarging the ion‐transfer channels and resulting in undesired ion contamination and reduced stability.^[^ ^130^, ^131^ ^]^ Similarly, denser pore structures in porous membranes enhance ion selectivity but decrease ion conductivity. Among different porous membranes, composite membranes have emerged as a promising choice for simultaneously improving ion selectivity and conductivity. Composite membranes consist of a porous support layer and a selective top layer, designed separately.^[^ ^132^ ^]^ Kim et al. reported a Nafion‐filled porous membrane for a flow ZBB, as shown in Figure 5c.^[^ ^133^ ^]^ The Nafion solution was cast on a porous polypropylene (PP) separator, and the Nafion ionomers were solidified, filling the pores during the drying process. The opaque PP separator transformed into a transparent one, indicating the success of the Nafion‐filled porous membrane. The PP fibrils in Figure 5d align along the in‐plane direction, and some observable pores are present. The Nafion phase filled in the pores of the PP separator effectively blocks the crossover of Br_2_. Additionally, Sun et al. developed a zeolite membrane with a porous framework within a zeolite molecular sieve to effectively confine the crossover/shuttle of soluble I_3_^−^.^[^ ^134^ ^]^ As shown in Figure 5e, I_3_^−^ easily diffuses to the anode surface during the charge process especially at low current rates (Figure 5f), causing overcharge, self‐discharge, and Zn corrosion. Confining soluble I_3_^−^ on the cathode side of the battery can effectively address these concerns. A simple and effective approach involves using a porous framework to effectively mesh the I_3_^−^ shuttle by the confinement of microporous structure. Compared with glass fiber (Figure 5f), the zeolite modified membrane exhibits improved CE from 78.9% to 98.6% at 0.2 A g^−1^ in the ZIB.

**Table** **2** presents the outstanding electrochemical performances of AZHBs based on various types of membranes, as reported previously.

**Table 2:** Comparisons of various membranes for AZHBs. MEP represents *N*‐ethyl‐*N*‐methylpyrrolidinium bromide. MEM represents 1‐ethyl‐1‐methyl morpholinium bromide. MEPBr represents 1‐ethyl‐1‐methylpyrrolidinium bromide.

Membrane	Electrolyte	Thickness [µm]	Area resistance [Ω cm^2^]	Permeability	Stability	Cell performance				Refs.
						Current density [mA cm^−2^]	CE [%]	VE [%]	EE [%]	
Nafion‐filled porous membranes	2.25 m ZnBr_2_ + 0.5 m ZnCl_2_ + 0.8 m MEPBr + 5 mL L^−1^ Br_2_	16	3.1	7.53×10^−7^ cm^2^ min^−1^	Stable in 166 cycling test	20	94.7	83.1	78.7	[133]
Porous polyolefin membranes	2 m KI + 1 m ZnBr_2_ + 2 m KCl	900	1.57	I_3_^−^: 7.11×10^−6^ cm^2^ s^−1^ I^−^: 4.53×10^−6^ cm^2^ s^−1^	Cycling more than 1000 cycles over 3 months	80	96	85	82	[135]
Prehydrated Nafion membranes	2.25 m ZnBr_2_ + 0.5 m ZnCl_2_ + 0.8 m MEPBr + 5 mL L^−1^ Br_2_	50	1.8	6.45×10^−6^ cm^2^ min^−1^	More than 350 cycles	20	85.4	88.4	75.4	[126]
Multiwalled carbon nanotubes/PAN‐Daramic membranes	3 m ZnBr_2_ + 3 m KCl + a 1:1 ratio of MEM and MEP	131.74	–	0.74 mmol L^−1^ h^−1^	Cycling more than 500 cycles	40	97	83	80.3	[136]
MEPBr‐Nafion daramic membranes	2 m ZnBr_2_ + 3 m KCl +0.4 m MEPBr	252.74	0.55	0.012 mmol L^−1^ h^−1^	Cycling over 120 cycles	40	97.4	87.5	85.3	[137]
Composite porous polyolefin ion conducting membranes	7.5 m KI +3.75 m ZnBr_2_	257	–	–	Run for more than 100 cycles	40	96	84.3	81	[138]

### 3.3 Zn Anodes

**Figure 6:**
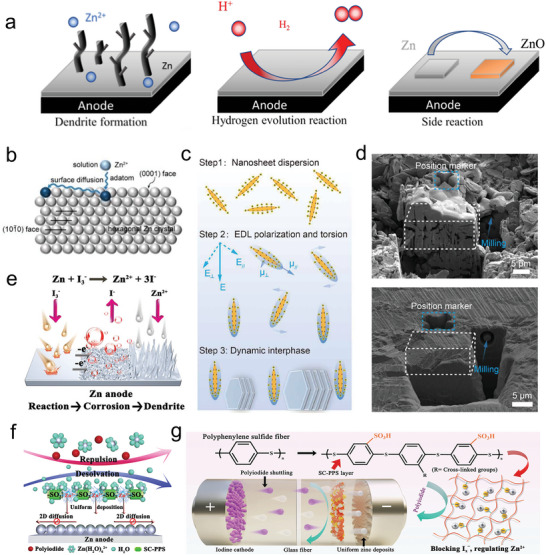
a) Schematic illustrating the fundamental challenges of Zn anode. Reproduced with permission.^[^ ^139^ ^]^ Copyright 2022, American Association for the Advancement of Science. b) Schematic illustration of hexagonal Zn crystal growth. Reproduced with permission.^[^ ^150^ ^]^ Copyright 2023, Wiley‐VCH. c) Schematic of the electro‐orientation process. d) Cross‐sectional SEM images produced by focused ion beam (FIB) reconstructions of Zn deposits in control 2 m ZnSO_4_ (aq) liquid electrolytes, and randomly oriented Zn electrodeposits assemble to form porous and loose microstructures. Reproduced with permission.^[^ ^155^ ^]^ Copyright 2021, American Association for the Advancement of Science. e) Schematic diagram for the mechanism of polyiodide corroding Zn anode. f) Schematic illustration for the brief synthesis process of SC‐PPS, as well as its roles in realizing high‐performance ZIBs. g) Schematic illustration of the stabilization of Zn anode with SC‐PPS layer. Reproduced with permission.^[^ ^154^ ^]^ Copyright 2023, American Chemical Society.

In neutral or weak acid electrolytes, metallic Zn with a moderate redox potential faces several challenges, such as the formation of Zn dendrites, hydrogen evolution, and side reactions (**Figure** **6**a).^[^ ^139^ ^]^ To address these persistent challenges, numerous intriguing strategies have been meticulously developed. Among these strategies, significant attention has been given to the modification of the anode/electrolyte interface (AEI), which encompasses the construction of the Zn anode surface and the regulation of the electrolyte composition.^[^ ^85^, ^140^ ^]^ This focus on AEI modification is warranted due to the fact that the AEI is the primary region where these challenges manifest.

From the perspective of the Zn anode, two specific approaches have shown promise in enhancing the reversibility of the Zn anode. First, the preferential exposure of the thermodynamically favorable (002) crystal plane has been demonstrated to be effective.^[^ ^141^, ^142^ ^]^ Second, the preconstruction of a SEI layer has also been proven to reinforce the reversibility of the Zn anode.^[^ ^143^, ^144^ ^]^

It is generally believed that adsorbed atoms will thermodynamically self‐diffuse into active sites within the lattice, such as steps, kinks, and grain boundaries.^[^ ^145^, ^146^ ^]^ For Zn (hexagonal close‐packed) crystals, adsorbed atoms on the (002) surface, which possesses the lowest surface energy, are unstable and tend to diffuse along the surface to other crystal planes, such as (100).^[^ ^147^, ^148^ ^]^ Therefore, it is thermodynamically favorable to form the (002) atomic plane. However, in practical solutions, dynamic microscale electro‐convective flows caused by surface irregularities (including substrate roughness and charge imbalances) lead to the formation of hotspots, resulting in the spontaneous deposition of Zn in locally aggregated forms under complex dynamics involving multiple transport fluxes.^[^ ^146^, ^149^ ^]^

Recently, we proposed a mechanism for spatial structural regulation of adsorbed molecules within the inner Helmholtz layer (IHP) to balance the kinetics of electrochemical reactions and the self‐diffusion of adsorbed atoms (Figure 6b).^[^ ^150^ ^]^ By adjusting the adsorption of solvated ions at the interface, we can effectively regulate the kinetics of electrochemical reactions, providing sufficient time for the self‐diffusion of adsorbed atoms, promoting layered growth, and creating conditions for preferred orientation. In this regard, we successfully reduced the adsorption energy of solvated zinc ions at the interface by introducing the adsorbed molecule tetramethylthiuram disulfide (TMS) with a ring‐shaped structure, resulting in a lower rate of electrochemical reaction kinetics compared to the self‐diffusion rate of adsorbed atoms. This approach enables the preferred orientation of hexagonal layered growth and (002) texture in Zn anodes, significantly improving CE and short‐circuiting time.

Furthermore, we dispersed g‐C_3_N_4_ nanosheets in the electrolyte, which enables the formation of an artificial dynamic interface (Figure 6c) and achieves high‐density (spatial density of ≈100%) and vertically aligned Zn electrodeposition (Figure 6d).^[^ ^151^ ^]^ The g‐C_3_N_4_ nanosheets exhibit high crystal matching and can dynamically adsorb/desorb on the (002) surface of Zn during the deposition/stripping process. In situ quantitative experiments demonstrate that ordered Zn deposition significantly reduces the consumption of Zn and the accumulation of by‐products within each cycle. Zhou et al. reported the construction of a polydopamine (PDA) layer on the surface of Zn, utilizing the diverse polar groups present in PDA to facilitate desolvation and ion confinement for [Zn(H_2_O)_6_]^2+^.^[^ ^152^ ^]^ This innovative approach ensures the uniform deposition of Zn^2+^ while effectively preventing the growth of Zn dendrites. Such dendrite suppression proves highly advantageous for enhancing battery performance. Additionally, other research endeavors have demonstrated the effectiveness of designing Zn anode coatings containing lead (Pb), denoted as Zn@Pb, which effectively shield the Zn substrate from corrosion reactions.^[^ ^153^ ^]^

When combined with a halogen cathode, these issues may be further amplified. Taking ZIBs as an example, Figure 6e illustrates the detrimental relationship between redox‐active species and Zn anodes, which can be described through three processes: i) the soluble I_3_^−^ pass through the membrane and react with Zn anode via shuttle effect, resulting in the formation of water‐soluble ZnI_2_; ii) Due to the presence of soluble I_3_^−^ and water corrosion, the surface of Zn foil gradually transitions from a flat to rugged structure, indicating the passivation occurred; iii) The formation of dendrites is uncontrolled due to the ultrairregular structure of the Zn anode, leading to rapid battery failure.^[^ ^154^ ^]^ Therefore, it is necessary to provide an effective protective layer for the Zn anode in AZHBs. Ensuring a uniform plating‐stripping process of the Zn anode and avoiding the shuttle effect of redox‐active species are both effective methods for protecting the Zn anode.

Lai et al. proposed the design of a sulfonate‐rich ion‐exchange layer (SC‐PSS) on the Zn anode, aiming to modulate the transport and reaction chemistry of polyiodide at the zinc/electrolyte interface, as illustrated in Figure 6f.^[^ ^154^ ^]^ Subsequently, Figure 6g demonstrated that the SC‐PSS coating effectively prevents the shuttle effect of I_3_^−^ due to the electrostatic repulsion generated by the electronegative sulfonic acid groups. Moreover, the hydrophilic sulfonic acid groups not only facilitate the desolvation of Zn^2+^, effectively mitigating water corrosion and other side reactions, but also contribute to the formation of a dense Zn layer to prevent dendrite formation. In addition to the SC‐PSS coating, the design of a fast Zn^2+^ conductive SEI layer effectively guides the Zn plating/stripping chemistry on the Zn anode. In the future, it is imperative to consider the influence of halogen cathodes and design the Zn anode accordingly.

### 3.4 Electrolytes

The electrolyte serves as the heart of the battery, acting as a bridge for ion transport and playing a crucial role in energy storage and release.^[^ ^156^ ^]^ The properties of the electrolyte, including solubility, ion conductivity, ESPW, and stability, significantly impact the performance and cycling stability of AZHBs.^[^ ^157^, ^158^ ^]^ An excellent electrolyte should ensure fast interface reactions while effectively reducing side reactions and halogen shuttle phenomena.

**Figure 7:**
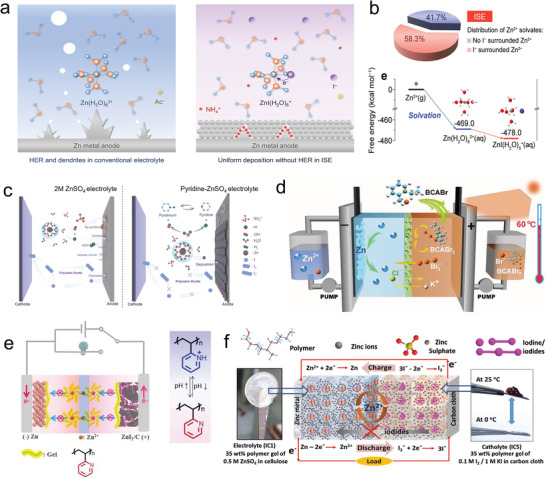
a) Design strategy of I^−^ participating solvation structure electrolyte. b) Distribution of the I^−^ participating solvation structure. Reproduced with permission.^[^ ^160^ ^]^ Copyright 2022, American Chemical Society. c) Schematic for mechanism for Zn–I_2_ full battery. For, left to right, respectively, 2 m ZnSO_4_ and pyridine‐ZnSO_4_ electrolyte. Reproduced with permission.^[^ ^161^ ^]^ Copyright 2023, Wiley. d) The scheme of a ZBFB with BCA as complexing agent. Reproduced with permission.^[^ ^70^ ^]^ Copyright 2021, American Chemical Society. e) Schematic illustration of the self‐protection function of stimulus‐responsive ZIBs. Reproduced with permission.^[^ ^164^ ^]^ Copyright 2020. American Chemical Society. f) Schematic representation of the working mechanism of ZIB with iodide diffusion control, top: chemical structures of component used in battery, left: digital image of electrolyte IC1 embedded in cellulose, right: digital image of IC5 catholyte at room temperature and homogenized in carbon cloth at 0 °C. Reproduced with permission.^[^ ^165^ ^]^ Copyright 2020, American Chemical Society.

As a stronger electron donor than water molecules, halogen ions prefer to coordinate with transition metal ions, leading to the replacement of water in the solvation structure of Zn(H_2_O)_6_^2+^.^[^ ^159^ ^]^ Chen et al. designed an electrolyte composed of zinc acetate and halide ammonium, where the electron‐donating anion I^−^ can coordinate with Zn^2+^, converting the traditional Zn(H_2_O)_6_^2+^ into ZnI(H_2_O)_5_^+^.^[^ ^160^ ^]^ The I^−^ can transfer electrons to water, suppressing the HER, as shown in **Figure** **7**a. Moreover, the type of anion (e.g., ZnCl_4_^2−^) will limit ion transport and Zn plating/stripping. As anticipated, Figure 7b demonstrates the optimal ratio of partial substitution of solvation structures with halogen ions, which can fulfill fast kinetics while reducing HER. However, there are still challenges to address, such as halogen diffusion and shuttle, leading to severe self‐discharge and low CE.

As shown in Figure 7c, Guo et al. reported a class of nitrogen‐containing heterocyclic compounds as organic pH buffer agents to suppress HER and anodic corrosion.^[^ ^161^ ^]^ These organic molecules with N‐based groups as hydrogen bond acceptors and donors interact with Zn^2+^ and iodine, regulating Zn deposition and inhibiting shuttle effects. Additionally, efforts have been made to study complexing agents that form larger complexes by binding with polyiodides and polybromides to suppress halogen crossover and shuttle effects.^[^ ^162^, ^163^ ^]^ As shown in Figure 7d, Li et al. designed a novel bromine complexing agent, 1‐ethyl‐2‐methyl‐pyridinium bromide (BCA), which exhibits a high EE of 84% even at 60 °C due to the strong interaction between BCA and polybromide anions.^[^ ^70^ ^]^

In Figure 7e, Feng et al. elucidated a stimulus‐responsive ZIBs by utilizing pH‐responsive electrolyte.^[^ ^164^ ^]^ The responsive mechanism trigged by the increase of electrolyte pH induced by HERs owing to overcharge. The pH‐responsive electrolyte based on poly(2‐vinylpyridine) (P2VP) can inhibit redox reactions under the overcharge state, consequent preventing the passivation reaction from Zn to ZnO at anode side and irreversible reaction from I_2_ to Zn(IO_3_)_2_ at cathode side. Thereby, the ZIBs possess capacity recovery of nearly 100%.

However, the aforementioned strategies lack the prevention of water‐soluble polyiodide formation, leading to self‐discharge during redox events. Therefore, Cui et al. presented a solid–gel reaction as depicted in Figure 7f.^[^ ^165^ ^]^ In this approach, a water‐based gel containing I_3_^−^/I^−^ is embedded in a block copolymer that carries highly active iodine components. The catholyte and electrolyte are separately incorporated into the polymer gel matrix formed by an amphiphilic block copolymer. The solid–gel interface facilitates the redox reaction, ensuring smooth zinc ion transport while preventing leakage of polyiodide anions.

## 4 Emerging Research Issues for AZHBs

### 4.1 Efficient Material Design

In order to enhance the performance and efficiency of batteries, the design of efficient materials is crucial. For the cathode, high‐performance materials with strong halogen adsorption/release capabilities can be selected to improve the electrochemical reaction rate and capacity. This can be achieved through surface engineering, control of porous structure, and appropriate additives. Additionally, the chemical stability of the cathode material can be optimized through synthesis methods and directed design. For the anode, it is important to design Zn electrode materials that are resistant to dendritic growth to reduce capacity loss caused by Zn dendrite short‐circuits.^[^ ^166^ ^]^ This can be achieved by controlling the crystal structure and interface engineering of the material. Furthermore, it is necessary to optimize the stability of the Zn electrode through material modifications, protective coatings, or alloying to suppress irreversible reactions and side reactions, thereby reducing capacity decay. As for the electrolyte, high ion transport is essential. Selecting an electrolyte with high ion transport rates can improve the charge/discharge rate and efficiency of the battery by optimizing the composition, concentration, and additives.^[^ ^167^ ^]^ Careful design of the electrolyte solvent structure can also be considered to maximize benefits and minimize drawbacks. Regarding the separator, designing low‐resistance separator materials is important to reduce internal resistance and improve power output and efficiency of the battery. This can be achieved by controlling the thickness, porous structure, and ion transport properties of the separator. Considering the crossover effect in ZBBs, designing separators with good ion selectivity can be achieved through optimization of the separator structure and the addition of functional layers.

During the material design process, it is necessary to consider the interactions among the Zn electrode, halogen cathode, electrolyte, and separator materials to achieve optimal performance matching and synergistic effects. Priority should also be given to selecting renewable and environmentally friendly materials to reduce reliance on rare resources and minimize environmental impact.

### 4.2 Thorough Mechanistic Analysis

**Figure 8:**
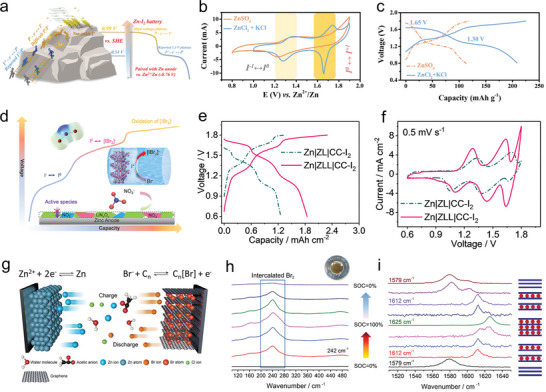
a) Illustration of the reactions of iodine batteries. Ti_3_C_2_I_2_//Zn battery based on optimized ZnCl_2_ + KCl electrolyte and conventional ZnSO_4_ electrolytes. b) CV curve at 10 mV s^−1^. c) Galvanostatic charge–discharge (GCD) curves at 0.5 A g^−1^. Reproduced with permission.^[^ ^68^ ^]^ Copyright 2020, Royal Society of Chemistry. d) Schematic for mechanism for quasisolid‐state zinc−dual halogen batteries. e) Voltage profiles of different battery systems at a current density of 3 mA cm^−2^. f) The corresponding CV curves of the full cells at a scan rate of 0.5 mV s^−1^. Reproduced with permission.^[^ ^157^ ^]^ Copyright 2022, American Chemical Society. g) Schematic diagram for the dual‐ion battery enabled by WSOE_45_‐1, PGA cathode, and Zn/GFF anode. h,i) Raman spectra (100–500 and 1510–1650 cm^−1^) of Br^−^ intercalated PGA cathode in situ during a charge–discharge cycle. Reproduced with permission.^[^ ^168^ ^]^ Copyright 2021, American Chemical Society.

Although we have provided an overview of the electrochemical reaction mechanisms of different halogen cathodes earlier, it is still necessary to conduct specific and detailed analysis of the electrode reaction mechanisms under practical conditions. For instance, under ideal circumstances, the oxidation‐reduction potential of iodine is 0.62 V versus SHE, corresponding to the redox process: I^0^ + e → I^−^, with a theoretical capacity of 211 mAh g^−1^.^[^ ^168^ ^]^ Recently, Zhi et al. achieved a series of multivalent state transitions in iodine cathode in ZIBs through electrolyte regulation strategies (**Figure** **8**a).^[^ ^68^ ^]^ The study demonstrates that F^−^ and Cl^−^ can activate and stabilize the transition of elemental iodine to higher oxidation states, which cannot be achieved in conventional ZIBs. In addition to the I^−^/I^+^ oxidation‐reduction occurring at 0.54 V, the new I^0^/I^+^ oxidation‐reduction is also fully activated. Consequently, two stable discharge plateaus exist in the battery, located at 1.7 and 1.45 V, respectively (Figure 8b,c), significantly enhancing the high potential output capability of the ZIB. Furthermore, they successfully developed a quasisolid‐state Zn‐dihalogen battery using an independent carbon cloth‐iodine cathode, a metallic Zn anode, and in situ prepared hydrogel electrolyte.^[^ ^157^ ^]^ In this well‐designed configuration, bromide ions in the hydrogel electrolyte form [IBr_2_]^−^ halogen species to stabilize the I^0^/I^+^ reaction, while the presence of iodine efficiently catalyzes the Br^−^/Br^0^ transformation in [IBr_2_]^−^ halogens. Consequently, a dual halogen conversion chemical reaction is realized on the electrode surface, involving three consecutive oxidation‐reduction reactions (i.e., I^−^/I^0^, I^0^/I^+^, Br^−^/Br^0^), as shown in Figure 8d. This conclusion is supported by charge–discharge curves (Figure 8e) and cyclic voltammetry (CV) tests (Figure 8f).

Gao et al. designed a dual‐ion halogen battery with self‐standing graphene (PGA) as the cathode.^[^ ^168^ ^]^ During charging (Figure 8g), Br^−^ is oxidized to nearly zero‐valent Br^0^, which is then embedded into the graphite lattice of the PGA cathode, forming C_n_[Br] graphite intercalation compounds (GICs). In situ Raman spectroscopy demonstrates the reversibility of the structural evolution of the PGA cathode during charge–discharge processes (Figure 8h,i). The absence of free Br_2_ Raman peaks during discharge indicates that the oxidized Br is almost entirely embedded into the graphite structure of the PGA cathode, rather than simply adsorbed on the electrode surface. During discharge, when the state of charge (SOC) reaches 0%, the Raman peak corresponding to the embedded Br gradually weakens or disappears, indicating the good reversibility of the Br embedding and extraction processes. These unconventional electrochemical mechanisms can further enhance the capacity advantages of AZHBs, thereby opening up possibilities for the diversified development of AZHBs and emphasizing the importance of detailed elucidation of electrochemical mechanisms.

### 4.3 Advanced Characterization

**Figure 9:**
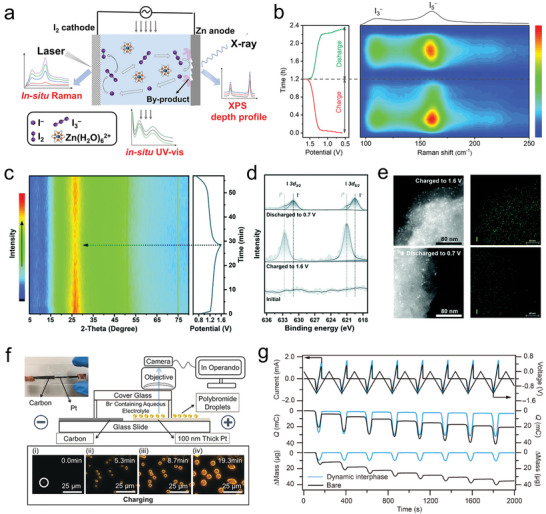
a) Schematic diagram revealing the estimation of the shuttle effect on the cathode, electrolyte, and anode components by in situ Raman spectra, in situ UV–vis, and XPS depth profiles, respectively. b) In situ Raman spectra, showing the electrochemical process of I^−^/I_2_ conversion in starch‐based ZIBs. Reproduced with permission.^[^ ^113^ ^]^ Copyright 2022, American Chemical Society. c) In situ XRD pattern of Zn‐AC/CFC battery during the first cycle. d) High‐resolution XPS I 3d spectra of AC/CFC cathode with 14‐05‐20 electrolyte. e) TEM‐EDX element mapping images at different charged and discharged states of AC/CFC cathode. Reproduced with permission.^[^ ^169^ ^]^ Copyright 2022, Royal Society of Chemistry. f) Schematic of the cell and microscopy for in operando visualization of bromine electrochemistry. The inset shows a real picture of the operando cell and time‐lapse dark‐field light microscopy images showing the formation of polybromide droplets. Reproduced with permission.^[^ ^170^ ^]^ Copyright 2019, Wiley‐VCH. g) Cyclic voltammetry (CV; 0.0 and −1.2 V vs Ag/AgCl), chronocoulometry, and EQCM tests to track the depositing/stripping process in different electrolytes at a scan rate of 10 mV s^−1^. Reproduced with permission.^[^ ^155^ ^]^ Copyright 2021, American Association for the Advancement of Science.

Advanced characterization techniques play a crucial role in understanding the reaction mechanisms of electrode materials, providing important guiding principles for designing high‐performance materials and optimizing battery systems. For instance, as shown in **Figure** **9**a, in situ Raman spectroscopy enables dynamic visualization of species changes during the iodine conversion reaction in ZIBs.^[^ ^113^ ^]^ In situ ultraviolet–visible spectroscopy (UV–Vis) allows real‐time detection of the concentration variations of multiple iodine ions in the electrolyte during cycling. Additionally, techniques such as X‐ray photoelectron spectroscopy/Auger electron spectroscopy (XPS/AES) can be employed to characterize the impact of shuttle effects on the Zn anode. Figure 9b displays the in‐situ Raman spectra of a specific ZIB conversion process, indicating that I_5_^−^ is the predominant conversion species, accompanied by the formation of a small amount of I_3_^−^ intermediates. After charging, the signals of I_3_^−^ and I_5_^−^ intermediates completely disappear, indicating the complete conversion of multiple iodine ions into I_2_. Similarly, after discharging, the signals of multiple iodine ions vanish, confirming the complete conversion of iodine. Zhou et al. characterized the reaction of the activated carbon electrode in ZIB using in situ X‐ray diffraction (XRD), as shown in Figure 9c.^[^ ^169^ ^]^ The intensity of the carbon characteristic peak (26.71°) weakens after complete charging and recovers during subsequent discharging, suggesting that the adsorption of I_2_ on the surface masks part of the signal and weakens the structural order of the activated carbon. However, due to its limited content, no diffraction peak indicating I_2_ adsorption is observed. Furthermore, they combined XPS for characterization. In the I 3d spectrum, two peaks associated with elemental I^0^ appear at 632.7 and 621.0 eV on the cathode, visually demonstrating the presence of adsorbed I_2_ on the cathode under complete charging conditions (Figure 9d). Additionally, transmission electron microscopy (TEM) also observed the presence of adsorbed I_2_ in the charged state (Figure 9e). Recently, Liu et al. designed an operational platform consisting of dark‐field optical microscopy and a transparent planar electrochemical cell to observe the nucleation and formation processes of polybromides in a flow ZBB (Figure 9f).^[^ ^170^ ^]^ These research findings successfully achieved microvisualization of polybromide formation and decomposition on the electrode, providing a new analytical approach for studying the mechanism of ionic liquid complexing agents in flow ZBBs. In the future, it is worth considering the development of characterization techniques that are sensitive to halogen species.

Recently, our team quantitatively characterized the deposition/stripping of Zn using electrochemical quartz crystal microbalance (EQCM) technology (Figure 9g).^[^ ^155^ ^]^ This technique can effectively detect the correspondence between charge and mass, thereby accurately assessing the deposition/stripping efficiency. In the future, it is encouraged to employ advanced characterization techniques for fine characterization of electrodes, electrolytes, and interfaces, especially through the use of in situ/ operando and combined techniques.

### 4.4 Performance Evaluation Under Operating Conditions

A comprehensive performance evaluation of batteries is crucial for understanding their energy conversion efficiency, cycle life, and other key performance parameters. EE is an important performance indicator of a battery, reflecting its ability to convert input energy into output energy. EE, as defined, is the product of CE and VE, as shown below

(21) $$\begin{array}{c} \mathrm{CE}=\left[\mathrm{Discharge}\;\;\mathrm{capacity}\left(\mathrm{Ah}\right)/\mathrm{Charge}\;\;\mathrm{capacity}\left(\mathrm{Ah}\right)\right]\times 100\% \end{array}$$

(22) $$\begin{array}{ccc} \mathrm{VE} & = & [\mathrm{Average}\;\;\mathrm{discharge}\;\;\mathrm{voltage}\left(V\right)/\\ & & \mathrm{Average}\;\;\mathrm{charge}\;\;\mathrm{voltage}\left(V\right)]\times 100\% \end{array}$$

(23) EE=CE×VE

Therefore, it is necessary to conduct a comprehensive evaluation of CE and VE, especially at different power densities.

Additionally, cycle life is one of the important indicators to assess battery performance. Cycle life refers to the ability of a battery to maintain its performance and capacity after multiple charge and discharge cycles. For flow batteries, cycle life is influenced by factors such as fluid flow, electrolyte decomposition, and electrode material corrosion.^[^ ^171^ ^]^ In performance evaluation, multiple cycle charge and discharge experiments are conducted, and the decay of battery capacity and the increase in internal resistance are monitored. By analyzing these data, the cycle life of the battery can be evaluated, and strategies for improving electrolyte composition, electrode material selection, and fluid dynamics design can be determined.

In addition to efficiency and cycle life, other performance parameters of the battery also need to be comprehensively evaluated. For example, power density, energy density, and stability of the battery are important parameters that have a significant impact on the performance and feasibility of the battery in different application fields. Through experimental testing and simulation analysis, these performance parameters can be determined and compared with other types of batteries for evaluation. Of note, performance evaluation is not limited to the measurement of a single parameter but requires a comprehensive consideration of multiple performance parameters. For instance, when evaluating the energy conversion efficiency of a battery, factors such as power density and cycle life also need to be taken into account. This holistic evaluation helps in gaining a comprehensive understanding of the performance characteristics of flow batteries and provides guidance for further optimization and improvement.^[^ ^172^ ^]^ Finally, we summarized the representative electrochemical indicators achieved by AZHBs in recent years, as shown in **Table** **3**.

**Table 3:** Summary of representative AZHBs.

Type	Cathode	Anode	Membrane	Electrolyte	Working potential [V]	Cyclic performance	CE [%]	EE [%]	Refs.
Flow ZBBs	Bimodal ordered mesostructured carbons	Carbon felt	Daramic HP with carbon ink	2 m ZnBr_2_ + 3 m KCl +0.4 m MEPBr	1.6	200 cycles at 80 mA cm^−2^	96.6	80	[98]
	Cage‐like porous carbon	Carbon felt	Daramic HP with carbon ink	2 m ZnBr_2_ + 0.4 m MEPBr	1.4	300 cycles at 80 mA cm^−2^	98	81	[104]
	TiN nanorods @ carbon felt	Graphite felt	Daramic HP	2 m ZnBr_2_ + 0.4 m MEPBr	1.5	100 cycles at 160 mA cm^−2^	97	80	[175]
	Nitrogen‐doped ordered mesoporous carbon	Graphite felt	Nafion‐212	2 m ZnBr_2_ +4 m NH_4_Cl	1.6	200 cycles at 80 mA cm^−2^	98	84	[101]
	Carbon felt‐based electrode with N‐rich defects	Graphite felt	Daramic HP	2 m ZnBr_2_ + 3 m KCl +0.4 M MEPB	1.4	140 cycles at 80 mA cm^−2^	≈99	65	[102]
	Protonated pyridinic nitrogen doped microporous carbon	Zn‐coated Pt	Membraneless	2.25 m ZnBr_2_ HBr to adjust the pH of 3.8.	1.6	1000 cycles at 20 mA cm^−2^	86	80	[87]
	Parasite Br_2_ to the Ti_3_C_2_T*_X_* MXene host	Zn	Glass fiber	1 m Zn(CF_3_SO_3_)_2_	1.5	2000 cycles at 2 A g^−1^	≈100	–	[111]

Type	Cathode	Anode	Membrane	Electrolyte	Potential working [V]	Cyclic performance	CE [%]	EE [%]	Refs.
Static ZBBs	Covalent organic framework (exCOF)‐bromine	Zn	Glass fiber	3 m ZnSO_4_	1.6	1000 cycles at 2 A g^−1^	99	–	[112]
	PBP‐BTAB‐HB polymer	Zn	Membraneless	ZnBr_2_	1.5	1200 cycles at 8 mA cm^−2^	92	75	[114]
	IBr @ carbon	Zn	Glass fiber	2 m ZnSO_4_	1.6	6000 cycles at 2 A g^−1^	95	–	[19]
	Carbon cloth	Zn	Glass fiber	Deep eutectic solvent	1.6	900 cycles at 0.3 mA cm^−2^	≈93	45	[176]
Flow ZIBs	Porous graphite	Porous graphite	Nafion‐117	6.5 m NH_4_I + 3.25 m NH_4_Cl	1.4	2500 cycles at 10 mA cm^−2^	≈99	78	[158]
	Nanoporous activated carbon fiber	Zn	Glass fiber	1.2 m ZnI_2_	1.3	Stable cycling after sweeping rate from 1 C to 133 C	92	78	[75]
	Graphite felt	Graphite felt	Nafion‐117	3.5 m ZnI_2_ + 1.75 m ZnBr_2_	1.3	50 cycles with 40 Ah L^−1^	≈95	75	[177]
	Graphite felt	Graphite felt	Nafion‐115	3.5 m ZnI_2_	1.3	40 cycles at 10 mA cm^−2^	≈99	82	[45]

Type	Cathode	Anode	Membrane	Electrolyte	Potential working [V]	Cyclic performance	CE [%]	EE [%]	Refs.
Static ZIBs	I_2_@ activated carbon −50	Zn	Glass fiber	2 m Zn(CF_3_SO_3_)_2_	1.2	10 000 cycles at 5 A g^−1^	99	96	[97]
	I_2_ encapsulated by hierarchical porous carbon	graphene‐ modified Zn foil	Glass fiber	2 m Zn(CF_3_SO_3_)_2_	1.1	35 000 cycles at 50 C	≈100	–	[95]
	I_2_‐Nitrogen‐doped porous carbon	Zn	Glass fiber	1 m ZnSO_4_	1.4	10 000 cycles at 10 C	≈100	–	[100]
	Nitrogen‐doped porous carbon nanocages/I_2_	Zn	Glass fiber	2 m ZnSO_4_	1.4	10 000 cycles at 0.1 A g^−1^	99	–	[88]
	B‐Fe‐NC/I_2_	Zn	Glass fiber	2 m ZnSO_4_	1.3	Over 10 000 cycles at 10 C	≈100	–	[103]
	Nitrogen doped porous carbons‐I_2_	Zn	Glass fiber	1 m ZnSO_4_ + 0.05 m ZnI_2_ + 0.05 m I_2_	1.3	Over 10 000 cycles at 1 A g^−1^	≈100	–	[178]
	Carbon cloth‐polypyrrole	Zn	Glass fiber	0.5 m ZnI_2_ +0.1 m HAc	1.3	600 cycles at 0.5 C	95.6	85	[110]
	Composed of mesoporous carbon and I_2_	Zn	Glass fiber	2 m ZnSO_4_	1.3	39 000 cycles at 10 A g^−1^	≈99	–	[179]

Type	Cathode	Anode	Membrane	Electrolyte	Potential working [V]	Cyclic performance	CE [%]	EE [%]	Refs.
	Carbon cloth−iodine composite cathode	Zn	Membraneless	3 m ZnSO_4_+3.5 m LiBr + 0.1 m LiNO_3_ aqueous gel electrolyte	1.7	500 cycles at 3 mA cm^−2^	97	–	[157]
	Encapsulated I_2_ in microporous carbon	Zn	Glass fiber	1 m ZnSO_4_	1.2	over 3000 cycles at 2 C.	≈100	–	[180]
	Starch	Zn	Glass fiber	Catholyte: 0.1 m I_2_ +0.1 m LiI Anolyte: 0.5 m ZnSO_4_ +0.5 m Li_2_SO_4_	1.3	50 000 cycles at 10 A g^−1^	≈100	–	[113]
	Co[Co*_x_* Fe_1−_ *_x_* (CN)_6_]/I_2_	Zn	Glass fiber	2 m ZnSO_4_	1.2	1500 cycles at 2 A g^−1^	≈100	–	[105]
	I_2_‐carbon composites	Zn	Glass fiber	1 m ZnSO_4_ + vermiculite nanosheets (VS)	1.2	40 000 cycles at 20 C	≈100	–	[85]
Static ZCBs	MnO_2_@carbon felt	Zn	Membraneless	5 mm MnSO_4_ +1 m ZnSO_4_ + 1 m LiCl	2.0	1000 cycles at 2.5 mA cm^−2^	92	–	[66]

### 4.5 Battery System Optimization

Battery pack design is an important aspect of battery system optimization. Optimizing the structure and layout of the battery pack can improve energy density, power density, and the stability of the battery system. A well‐designed series and parallel configuration of the batteries can balance the pressure differences among the batteries and enhance the overall performance of the system. Optimizing these systems is crucial for improving their performance, efficiency, and overall reliability.^[^ ^173^ ^]^ The control strategies for optimizing the battery system can enhance the operational efficiency and energy utilization of the system. Depending on different application scenarios, appropriate charging and discharging strategies and power regulation algorithms can balance the energy demand and the performance characteristics of the batteries. For flow battery systems, it also involves the design and flow configuration of the batteries. The design of channels and electrodes affects fluid distribution, material transport, and overall system efficiency. Additionally, precise control of flow rate and electrolyte management can minimize pressure drop, improve mixing efficiency, and enhance energy conversion efficiency.

Efficient control and management of both static and flow battery systems are crucial for achieving optimal performance. Adopting advanced battery management systems (BMS) to monitor battery cell voltage, temperature, and state of charge/discharge can optimize charging and discharging strategies, prolong battery life, and ensure safe operation.^[^ ^174^ ^]^ Intelligent algorithms and predictive models can be used to optimize energy allocation and scheduling, taking into account factors such as power demand, pricing, and integration of renewable energy sources.

## 5 Summary and Perspective

In this review, we analyze the research background, development history, fundamental chemistry, and scientific issues of ZHBs. We provide a systematic introduction to current solutions, particularly at the material level. Additionally, we review the key electrochemical metrics achieved through various strategies and evaluate the diverse optimization approaches and research directions for achieving high‐performance AZHBs.

**Figure 10:**
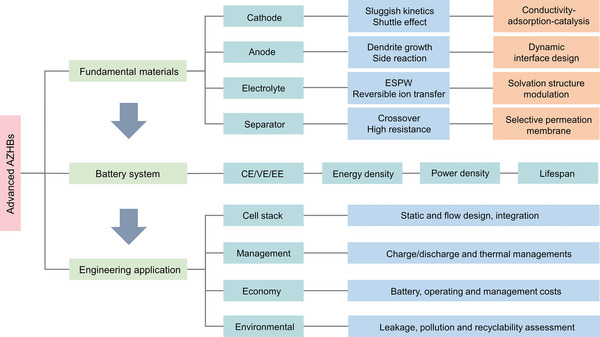
A chart diagram illustrates the fundamental materials, battery system, and engineering application of AZHBs.

Despite notable strides made in the realm of AZHBs, a plethora of formidable challenges beckon resolution. When juxtaposed against their conventional energy storage counterparts, the industrialization of AZHBs confronts substantial impediments encompassing underwhelming electrochemical performance metrics, prohibitive manufacturing expenses, as well as questions of stability and reliability, among a myriad of other considerations. Prospective research endeavors in this domain are poised to embark on a multifaceted trajectory, delineated across three progressively nuanced dimensions: fundamental materials, battery system, and engineering application, as summarized in **Figure** **10**.

### 5.1 Fundamental Materials

Addressing the foundational material conundrums at the heart of AZHBs, myriad investigations have sought to optimize pivotal constituents, such as cathodes, anodes, electrolytes, and separators, in the quest for large‐scale commercialization. However, adopting a unilateral approach is but a partial solution. Concerning cathodes, the pernicious shuttle effect, stemming from the genesis of polyiodides and polybromides, precipitates a diminution in CE and discharge capacity. Concurrently, the ponderous kinetics of halogen element conversion reactions pose a substantial hurdle. Consequently, the development of superior materials hinges on the optimization of strategies pertaining to conductivity, adsorption, and catalysis. Furthermore, the achievement of uniform Zn metal deposition and the suppression of ancillary reactions constitute vital avenues to redress anode‐related issues and protract the life expectancy of AZHBs. Although artificial SEI layers have been introduced to safeguard zinc anodes and curtail dendritic growth, the longevity of artificial SEIs during protracted cycling remains uncertain. Hence, the exploration of dynamically self‐repairing interfacial configurations emerges as a prospective panacea. Moreover, the judicious modulation of Zn^2+^ solvation structures promise thermodynamic and kinetic control over ancillary reactions and deposition/stripping processes. In light of the shuttle effect, the optimization of membrane selective permeability assumes paramount significance. Guiding membrane design principles revolve around impeding the penetration of polyiodides and polybromides while preserving robust ion conductivity. Forthcoming strategies may encompass membrane pore engineering, structural refinement, surface/internal chemical modification, among other avenues, to yield membranes characterized by heightened selective permeability.

### 5.2 Battery System

At the battery system stratum, the appraisal of performance parameters can be categorized into a multitude of dimensions encompassing CE/VE/EE, energy density, power density, and cycle life. These aspects are inherently contingent upon fundamental materials, mandating the confluence of disparate elements and strategies to navigate the crucible of AZHB commercialization. Moreover, the cultivation of pragmatic evaluations of AZHB performance remains an imperious endeavor. In the realm of static batteries, parameters such as the negative/positive (N/P) ratio, electrolyte volume, and active material loading warrant further optimization. For flow batteries, the concentration/environment of the electrolyte and the coupling of power and capacity assume outsized significance as arbiters of performance feedback.

### 5.3 Engineering Application

The design and integration of cell stacks are pivotal factors influencing the performance and efficiency of AZHBs. Future developments will focus on enhancing the efficiency and scalability of these batteries. Optimization of static and flow designs will play a critical role in increasing energy and power densities, making them suitable for diverse applications. Additionally, advanced integration techniques will reduce the overall system footprint, enhance overall performance, and lower maintenance costs. Effective battery management is paramount to ensure the long‐term stability and reliability of AZHBs. Future developments will emphasize the advancement of charge/discharge management technologies to maximize battery lifespan. Furthermore, improvements in thermal management systems will help maintain battery performance across various operating temperatures, enhancing durability. Smart management systems will provide real‐time monitoring of battery status, optimize charge/discharge strategies, and proactively identify potential issues, thereby increasing the reliability and efficiency of AZHBs. As competition in the battery market intensifies, cost reduction is a key driver for the widespread adoption of AZHBs. Future developments will focus on lowering manufacturing costs, increasing energy efficiency, and reducing maintenance expenses. Researchers will strive to identify cost‐effective materials and production processes while adopting sustainable manufacturing methods. Moreover, advanced battery management systems will further optimize performance, lower operating costs, and enhance competitiveness in the market. Economic viability will be a critical factor in determining the success of AZHBs in commercial applications. Environmental sustainability is also a crucial consideration in the development of AZHBs. Future research efforts will be directed toward minimizing the risk of leakage within battery systems and reducing environmental pollution of halogen species. This includes enhancing the stability and reliability of battery materials to prevent hazardous substance leakage. Furthermore, assessments of recyclability and reusability will become paramount in reducing the environmental footprint of spent batteries. Adherence to circular economy principles will play a larger role in battery design and production, ensuring the sustainable utilization of resources.

In conclusion, AZHBs are poised to play a pivotal role in the future of energy storage. Through ongoing engineering innovations and research developments, we can anticipate more efficient, reliable, cost‐effective, and safer AZHBs technologies. These advancements will help meet the growing demands of renewable energy integration, and grid energy storage, ultimately driving the development of clean energy and a sustainable future.

## Conflict of Interest

The authors declare no conflict of interest.
